# Empirical analysis of influencer attributes and social satisfaction effects on purchase intentions in chinese social media

**DOI:** 10.1038/s41598-025-03336-6

**Published:** 2025-05-29

**Authors:** Yifan Yao, Dongxia Meng, Xiaoguang Wei

**Affiliations:** Fintech Department, Hebei Finance University, 3188 Hengxiang N Ave, Bei Shi Qu, Baoding, Hebei 072556 China

**Keywords:** Influencer marketing, Attribution theory, Confirmatory factor analysis, Structural equation modeling, Mediation analysis, Moderation analysis, Social media, Psychology, Mathematics and computing

## Abstract

Underpinned by attribution theory and source credibility theory, this study investigates how influencer characteristics and customer’s prior product knowledge affect purchase decisions in the context of social media marketing. A conceptual model incorporating nine potential antecedents was developed based on identified research gaps. Confirmatory factor analysis (CFA) and structural equation modeling (SEM) were conducted using data from an online survey of 363 respondents who follow entertainment-type influencers. Results reveal that social satisfaction mediates the relationship between influencer characteristics and purchase intention, while customers’ product knowledge moderates this mediated relationship. Specifically, visual aesthetics and denotative inspiration significantly influence social satisfaction, whereas influencer level and connotative inspiration show no significant effects. The study contributes to the theoretical understanding of influencer marketing by integrating attribution theory in a digital context, particularly within the Chinese market. These findings offer practical insights for businesses and marketers in optimizing influencer selection and content strategies, with particular relevance for the rapidly evolving Chinese social media landscape.

## Introduction

### Overview

The development of social media has revolutionized the way consumers acquire product information compared to traditional methods^1^. Social media influencers are individuals with substantial followings across multiple social media platforms^2–4^. Consequently, influencer marketing has emerged as a strategy where brands and consumers achieve their respective marketing and purchasing goals through the influence of online social media personalities^5,6^. According to the China Internet Network Information Center (CINIC), as of 2020, there were over 904 million Internet users in China. Moreover, the explosive growth of social media platforms, such as WeChat and Weibo, has led to 1.04 billion active Chinese social media users, maintaining at least one social media account^7–9^. This trend highlights the unique research opportunities presented by the Chinese influencer marketing context.

The transformation of social media influencers into Key Opinion Leaders (KOLs) and Professional Generated Content (PGC) creators has profoundly influenced both consumer behavior and brand strategies in the digital age. This paradigm shift has disrupted conventional advertising models, fostering a consumer journey characterized by heightened interactivity and participation. Simultaneously, the proliferation of information sources has empowered consumers with enhanced analytical capabilities, enabling more sophisticated assessments of influencer authenticity and content quality^10,11^.

Against this backdrop, the present study investigates the evolving dynamic between influencers and consumers by examining how specific influencer attributes and the nature of follower engagement affect purchase intentions. By identifying the key antecedents influencing this relationship and proposing a comprehensive conceptual framework, this research seeks to offer novel insights to inform and optimize influencer marketing strategies.

### Research motivation

The rapid rise of digital marketing and social media influencers has transformed conventional advertising frameworks^10^. Recent scholarship has highlighted the need for greater insight into consumer behavior within digital environments^12,13^, yet research applying attribution theory specifically to influencer marketing, particularly in emerging markets such as China, is scarce.

Prior studies often address influencer characteristics in generalized terms. For example, Lou and Yuan^10^ developed a framework linking trust in influencer-created brand content to brand awareness and purchase intention, categorizing trust into dimensions such as informational value, entertainment value, trustworthiness, attractiveness, and similarity. While informative, these broad constructs do not provide an in-depth examination of specific influencer attributes. Similarly, Jin, Muqaddam, and Ryu^14^ proposed a model involving trustworthiness, brand attitude, and envy related to social presence, but their study also lacked granularity regarding influencer characteristics and content. This limitation has been noted in the literature, with calls for more nuanced research approaches^15^.

To address these gaps, our study proposes a detailed investigation of influencer characteristics, including educational background, socioeconomic status, and influencer tier. In addition, given the prevalence of short-video platforms^16^, the analysis incorporates content-specific elements. The distinctive dynamics of China’s social media landscape-with over 904 million internet users and 1.04 billion active accounts, yet systematic research on the effects of these market conditions on influencer marketing effectiveness remains limited.

Moreover, while relationships between influencer characteristics and purchase intentions have been studied, the mediating role of social satisfaction and the moderating effect of consumers’ product knowledge remain underexplored, despite increasing user sophistication and product expertise diversity.

This study aims to bridge these gaps by applying attribution theory to influencer marketing in the context of Chinese social media. It examines specific influencer attributes and content factors rather than relying solely on broad classifications. Furthermore, the research explores how mediating and moderating factors, such as social satisfaction and product knowledge, impact purchase intentions. By doing so, this study contributes to both academic understanding and practical strategy development within influencer marketing.

In summary, this research advances the application of attribution theory in digital marketing by focusing on the influencer marketing sector within emerging markets like China. It responds to calls for granularity by investigating specific influencer and content attributes, as well as the mechanisms that shape influencer marketing effectiveness.

### Research contribution

Following Whetten’s^17^ framework, this study provides several key theoretical contributions. First, it advances influencer marketing theory by disaggregating influencer characteristics into distinct dimensions-such as educational background and socioeconomic status instead of treating these factors as homogeneous constructs. Second, it underscores the relevance of content attributes (e.g., visual aesthetics, denotative and connotative inspiration) as distinct mechanisms of influence. Third, it establishes social satisfaction as a critical mediating mechanism in the relationship between influencers and purchase outcomes. This study also uncovers previously unexamined dynamics within influencer marketing. For example, it finds that visual aesthetics and denotative inspiration directly promote social satisfaction, whereas influencer tier and connotative inspiration do not. Importantly, the research demonstrates a dual-process pathway wherein social satisfaction mediates the link between influencers and purchase intentions, with customer product knowledge moderating this effect.

This research enhances theoretical understanding in two principal areas. First, it integrates attribution theory with source credibility theory to account for why certain influencer attributes and content features are more effective in driving purchase intentions. Second, it sheds light on the psychological mechanisms by which social satisfaction translates influencer characteristics into concrete consumer behavior. Examining these relationships in the context of China’s rapidly evolving social media landscape, the study broadens the application of attribution theory to new digital marketing settings. The study’s cultural context deepens the theory’s cross-cultural relevance and lays the groundwork for comparative research in broader markets.

From a practical standpoint, the findings indicate that socioeconomic status significantly impacts social satisfaction and subsequently purchase behavior. Marketers should therefore select influencers whose socioeconomic profiles align with brand positioning, rather than relying solely on follower metrics. In light of Chinese consumers’ preference for visually engaging and practical (denotative) content, marketing collaborations should emphasize these aspects over emotional or ambiguous messaging. Additionally, businesses are advised to use social satisfaction as a key performance indicator (KPI) for influencer campaigns, given its central mediating role; efforts should focus on fostering community and authenticity to maximize social satisfaction. Finally, the salience of socioeconomic status in the Chinese context highlights the importance of a culturally informed approach. Brands entering the Chinese market should avoid simply adopting Western influencer models and instead tailor their strategies to local conditions.

## Literature review

### Theoretical review

There are four main theories primarily applied to influencer marketing topics: source credibility theory, persuasion knowledge model theory, social comparison theory, and attribution theory. Source credibility theory posits a relationship between influencers’ characteristics and audiences’ acceptance of information^18^. Researchers have applied this theory to examine how varying degrees of influencer credibility affect the effectiveness of endorsements in shaping followers’ purchase intentions and attitudes^19^. Friestad and Wright introduced the persuasion knowledge model (PKM) in 1994^20^. Subsequent studies have employed the PKM to explore the impact of consumers’ persuasion knowledge,particularly following sponsorship disclosures on their responses to sponsored posts^21,22^. Social comparison theory, initially proposed by Festinger^23^, suggests that individuals assess their values and abilities by comparing themselves to others.

Attribution theory serves as a theoretical framework for explaining causal relationships between consumers’ purchase intentions and influencers’ actions^24^. Key aspects of attribution theory include internal and external attribution. Internal (dispositional) attribution assigns behavior to personal traits, abilities, or efforts; external (situational) attribution links it to environmental influences or circumstances. In traditional marketing contexts, Folkes^25^ applied attribution theory to consumer purchase behavior, focusing on how consumers evaluate product risk and using multiple attribution dimensions (stability, controllability, and globality) rather than solely internal versus external attribution. Kim, Balasubramanian, and Fiore^26^ extended this theory to investigate how digital marketing channels affect consumer purchase decisions, developing a model that quantifies the impact of different digital marketing channels including display advertising, paid search, and email marketing on purchase outcomes. This approach marked a novel application of attribution theory, broadening its scope from psychological explanation to evaluating marketing effectiveness.

Unlike traditional attribution theory, which centers on how individuals interpret behavioral causes through internal (e.g., ability, effort) and external (e.g., luck, environment) factors, recent research has adopted the concept of “marketing attribution.” This extension enables a data-driven assessment of how specific marketing touchpoints contribute to final conversions, bridging theoretical frameworks and practical strategies within the digital ecosystem. Jhawar et al.^27^ identified critical issues such as content authenticity, consumer vulnerability, and unintended consequences of influencer strategies. These findings align with attribution theory, highlighting that perceptions of influencer authenticity and credibility can be shaped by contextual factors. Building on this, our study offers a more nuanced perspective by examining how specific influencer characteristics (e.g., educational background and socioeconomic status) and content attributes (such as visual aesthetics and types of inspirational content) influence consumer outcomes. This approach addresses both the benefits of influencer marketing and potential drawbacks, especially regarding consumer vulnerability and authenticity perceptions.

Kumar et al.^28^ provided a comprehensive review of artificial intelligence applications in marketing, with particular attention to attribution modeling. Departing from traditional attribution theory,which emphasizes the internal-external dichotomy in individual behavior,this research explores AI-driven multi-touch attribution within digital marketing. Rather than adopting a classical psychological viewpoint, the focus is on quantifying the impact of marketing touchpoints on consumer decisions through machine learning algorithms analyzing complex customer journeys. This represents an evolution in attribution theory’s application, shifting from understanding individual cognition to optimizing resource allocation in marketing.

Kannan and Li^12^ presented a nuanced application of attribution theory in digital marketing, arguing that advanced multi-touch attribution models-rather than classic last-click attribution-should be used to explain consumer purchase behavior in social media marketing. However, several studies have noted limitations in applying attribution theory to social media. For example, Voramontri and Klieb^13^ highlighted the complexity of multi-platform environments, characterized by real-time engagement and personalized experiences, which challenge the static structure of conventional attribution models. The influence of social networks and peer effects further complicates attribution, going beyond the scope of individual-centric models^29^. Additionally, the cross-device consumer journey and contextual specificity of social media use introduce further variables not easily accommodated by existing frameworks^30^. These limitations underscore the need for more sophisticated, flexible, and holistic attribution models to address the nuanced dynamics of consumer behavior within the social media ecosystem.

Although there are some theory limitations applied in influencer marketing, recent advancements in attribution theory have significantly addressed its limitations in the complex digital marketing landscape. Li and Kannan^31^ pioneered a comprehensive multi-channel attribution model, incorporating cross-device interactions, while Abhishek, Fader, and Hosanagar^32^ developed a multi-stage framework capturing both long-term and short-term effects throughout the consumer decision journey. Berman^33^ leveraged machine learning techniques to tackle the intricacies of multi-touch attribution, enhancing the model’s adaptability to diverse digital environments. Anderl et al.^34^ introduced graph-based modeling to visualize and analyze intricate cross-platform user behaviors, offering improved insights into the customer journey. Furthermore, Ghose and Todri-Adamopoulos^35^ integrated ad characteristics and browsing behaviors into a dynamic attribution model, accounting for the temporal and spatial aspects of digital advertising. These studies collectively address key challenges by integrating multi-channel and cross-device behaviors, implementing dynamic models to capture real-time interactions. In addition, studies use advanced machine learning algorithms for complex data analysis and incorporated personalised user patterns.

Unlike previous studies that mainly focused on general influencer characteristics (e.g., Lou and Yuan’s^10^ trust dimensions, Jin et al.’s^14^ credibility factors), this study provides a more granular analysis examining specific personal attributes (education background, socioeconomic status) and analyzing content-specific factors in short-video context (visual aesthetics, denotative and connotative inspiration). Also, the study will introduce customer product knowledge as a key moderating variable. Moreover, this study will extend attribution theory in Chinese digital marketing context by examining how Chinese consumers attribute influencer success to internal factors (education, socioeconomic status) versus external factors (platform algorithms). Investigating how cultural values (e.g., emphasis on education and social status) affect attribution processes is also an extension.

### Empirical review

Recent empirical studies on influencer marketing have employed various methodologies, revealing valuable insights while also revealing methodological limitations. Previous studies concluded that most academic research on influencer marketing primarily applies quantitative methods based on empirical research^14,18,36–38^. The confirmatory factor analysis (CFA) and the structural equation modeling (SEM) are always applied in previous studies^9,39,40^. CFA is also a technique in testing the measurement model^41^. Besides, bootstrapping-based resampling is conducted to examine the structural validity of the model, which also serves as hypotheses testing. It is important to note that most studies only sample from a single source of consumers. For example, Amazon Mechanical Turk (MTurk) is a crowdsourcing platform developed by Amazon. MTurk’s data is reliable^42,43^, but data sampling still has some limitations. For instance, there may be non-English-speaking participants on MTurk, which means conclusions cannot always be generalized to specific country populations. The study^44^ explored how customers engaged in travel social influencers on social media. The focus was on understanding the dynamics of customer engagement with travel social influencers on social media platforms. The research explored customers’ engagement with influencers by examining different influencers’ characteristics. There are 9 characteristics identified in the study, including credibility, expertise, and attractiveness, which are considered from a situational perspective. Other factors belong to the personality perspective. The paper also established a conceptual model with a two-subject design method based on situational and personality perspectives. The innovation of this study is that it brings two perspective views in the study, providing a comprehensive understanding of the influencer-customer relationship. Lou and Yuan^10^ conducted an online survey (N = 297) among Instagram users, employing Structural Equation Modeling (SEM) to investigate the impact of influencer content value and credibility on consumer brand awareness, trust, and purchase intentions. Their findings indicated significant positive effects of informational value, entertainment value, and credibility on brand awareness, as well as positive influences of influencer expertise, attractiveness, and similarity on brand trust. However, the study’s generalizability is limited by its focus on a single platform (Instagram), cross-sectional design, and reliance on self-reported data. In a more extensive cross-platform comparison, Sokolova and Kefi^45^ surveyed 1,209 participants across YouTube and Instagram. Utilizing Partial Least Squares Structural Equation Modeling (PLS-SEM), they found that on YouTube, influencers’ physical attractiveness and attitude homophily significantly affected purchase intentions, while on Instagram, social attractiveness and attitude homophily were more impactful. Despite the larger sample size, this study was constrained by its focus on only two platforms and potential cultural biases.

In conclusion, future research in influencer marketing should address several key methodological considerations. Firstly, there is a pressing need for more diverse research methodologies, particularly longitudinal studies and mixed-method designs. These approaches would enable researchers to capture the long-term effects and complex dynamics of influencer marketing, providing a more comprehensive understanding of its impact over time^46,47^. Secondly, expanding the scope of sampling to include a wider range of demographic characteristics and cultural backgrounds is crucial. This expansion would enhance the generalizability of research findings, allowing for more robust and universally applicable conclusions^10,48^. Thirdly, integrating actual purchase data with social media interaction metrics could significantly improve the accuracy of assessing influencer marketing effectiveness. This data triangulation approach would provide a more realistic evaluation of the tangible outcomes of influencer campaigns, moving beyond self-reported intentions to actual consumer behaviors^14,45^.

### Conclusion of the literature review

Based on previous studies^45,49^, several gaps remain in influencer marketing research. These include not only the limited application of attribution theory in digital marketing contexts, but also inadequacies in conceptual model design. Most prior models have relied on general segmentation factors when examining influencer marketing, rather than focusing on more specific influencer attributes. In contrast, this research seeks to examine distinct characteristics of influencers, such as education, socioeconomic status, and influencer tier in greater detail than previous studies. Furthermore, within the context of short-video social media platforms, the effects of influencers’ content attributes on consumers’ final purchase decisions are underexplored. The literature also rarely considers whether consumers’ knowledge of influencer-marketed products influences their purchase decisions, an issue that will be addressed specifically in the hypothesis development. Therefore, this study incorporates customer product knowledge as a moderating factor in the influencer marketing model.

## Hypothesis development

### Model construction logic

**Fig. 1:**
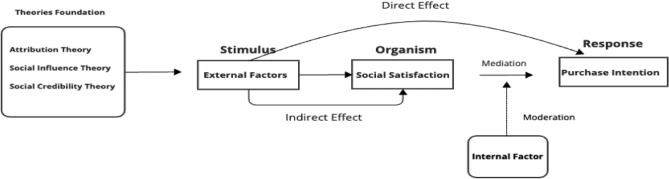
Model logic.

The research model is constructed based on three theoretical foundations and practical considerations. Its structural relationships draw from attribution theory^24^ and prior empirical research. First, the direct effect of influencer attributes on purchase intention is supported by evidence that source characteristics directly influence consumer behavior^10^. The link between social satisfaction and purchase intention is grounded in social influence theory^50^. Second, social satisfaction is positioned as a mediator, reflecting its role as the psychological mechanism through which influencer characteristics impact purchase decisions; prior studies have shown that satisfaction mediates the relationship between source traits and behavioral intentions^51^. Third, product knowledge is incorporated as a moderator, as consumer expertise influences information processing and the importance placed on various information sources^52^. Figure 1 illustrates this decision-making process.

### Education

Black et al.^53^ found that education is positively related to credibility. The education antecedent is an essential factor explaining customers’ perceived credibility in brand advertising^54^. Educated influencers are likely to possess greater expertise in their field, which may enhance their persuasiveness under the persuasion knowledge model (PKM). Extending this idea to influencer marketing, influencers with higher educational qualifications may be perceived as more credible and thus exert greater influence on consumer behavior. However, most existing research has focused on the role of customer education, rather than the education level of influencers themselves^55^. Consequently, there is limited evidence on how influencer education moderates consumer purchasing decisions. Grounded in source credibility theory, this study considers influencers’ education as a key factor, with respondents assessing influencers rather than themselves as customers, to help fill this gap. Thus, the following hypothesis is proposed:

**H1:** influencers’ education status is positively related to customer social satisfaction.

### Socioeconomic status

Researchers have examined various aspects of socioeconomic status-such as income and occupation to assess their influence on consumer attitudes and interactions with influencers or brands. Hati and Idris^56^ found that customers’ socioeconomic status affects their attitudes toward enterprises. With respect to influencers, Shin and Lee^57^ showed that influencers with higher socioeconomic status can prompt consumers to engage in intentional mimicry consumption out of curiosity. However, there is limited research on how an influencer’s socioeconomic status affects followers’ social satisfaction. Given this gap, further investigation is needed to clarify how socioeconomic status shapes not only consumer behavior but also the social dynamics between influencers and their audiences. For example, Lee and Watkins^58^ found that higher socioeconomic status enhances the perceived authenticity of influencers, thereby increasing follower trust and satisfaction. Similarly, Ali, Tretiakov, and Whiddett^59^ argued that transparent communication of an influencer’s background and lifestyle can foster a stronger sense of community and enhance social satisfaction. Despite these insights, existing research on the impact of influencer socioeconomic status on social satisfaction remains limited. Therefore, following previous findings and drawing on source credibility theory, this study proposes the following hypothesis:

**H2:** influencers’ socioeconomic status is positively related to customer social satisfaction.

### Influencer level

The concept of influencer level has emerged as a critical dimension in social media marketing research^60^. Influencer level refers to the hierarchical classification of social media influencers based on quantitative and qualitative metrics, including follower count, engagement rate (likes, comments, shares), and influence index^61,62^. Based on follower count, influencers are generally categorized as macro (typically over 100,000 followers) or micro (typically between 1,000 and 100,000 followers). This study focuses on influencer level for several reasons. First, influencer level is a more objective and measurable indicator than subjective attributes such as perceived ability or expertise. Second, in China’s rapidly evolving social media landscape, where platforms like WeChat, Weibo, and Douyin have over one billion active users^7^, understanding the impact of influencer level is crucial for developing effective marketing strategies. Third, the relationship between influencer level and marketing effectiveness is still debated, with some studies suggesting that micro-influencers may achieve higher engagement despite their smaller follower base. Recent research has highlighted the distinctive role of influencer level in social media marketing. For example, Lou and Yuan^10^ found that influencer level influences followers’ trust and purchase intentions through mechanisms different from those associated with other influencer characteristics. Similarly, Sokolova and Kefi^45^ demonstrated that the effectiveness of influencer marketing varies significantly by influencer level, indicating that a larger follower base does not necessarily result in greater influence or follower satisfaction. These findings underscore the need to examine influencer level as an independent factor in social media marketing effectiveness.

Drawing on attribution theory, influencer level is hypothesized to be negatively correlated with social satisfaction. The theoretical rationale is that, when engaging with high-level influencers, consumers may attribute their success to external factors, such as platform algorithms or commercial partnerships, rather than to personal ability or authenticity. This external attribution may diminish perceived affinity and authenticity, thereby reducing social satisfaction. Conversely, consumers interacting with lower-level influencers are more likely to attribute success to personal effort and authenticity, thus fostering a stronger sense of social connection.

**H3:**: Influencer level is negatively related to social satisfaction.

### Visual aesthetics

Visual content on social media platforms is shorter than traditional movies. Influencers can edit them with the help of software functions, including adding filters, special effects, and subtitles. In the shooting and post-production of short videos, the creator adds digital technology, which brings a different visual experience to the audience with a deep impression. The significant role of visual aesthetics of websites has been emphasised in the e-commerce context^63,64^. Results^65–67^ show that having a visual aesthetic should form an essential component of Instagram sales conversion strategy for online retail businesses. Thus, according to previous studies, Visual aesthetics is considered to apply in the model. Based on the source credibility theory, H4 is proposed:

**H4:** influencers’ content visual aesthetic is positively related to customer social satisfaction.

### Connotative inspire and denotative inspire

Consumers may attribute the inspirational nature of influencer content either to the influencer’s knowledge and insights (internal attribution) or to external information sources (external attribution). Both attribution processes can potentially impact social satisfaction. Research on connotative and denotative meanings is primarily situated within the field of advertising. Specifically, denotative meaning refers to the literal meaning, while connotative meaning is evaluative and emotional^68^. In semiotics, these are termed denotation and connotation, while in psychology, they are referred to as lexical and psychological meanings^69^. In consumer research, these concepts are defined as attribute and performance dimensions^70^, comprehension and interpretation^71^, or, more generally, as ‘recognition/identification’ and ‘interpretation.’ Previous research on how influencers’ use of denotative and connotative meanings relates to consumers’ purchase intentions is limited. Based on the persuasion knowledge model (PKM) and source credibility theory, the following hypotheses are proposed:

**H5:** influencers’ connotative inspire quality is positively related to customers’ social satisfaction.

**H6:** influencers’ denotative inspire quality is positively related to customers’ social satisfaction.

### Social satisfaction with purchase intention

Social satisfaction refers to the emotional fulfilment, sense of belonging, and social identity that users gain from social media interactions. In the context of social media marketing, it encompasses the positive emotional experiences users have after engaging with a brand or influencer^72^. Higher levels of social satisfaction can enhance user engagement and encourage positive word-of-mouth. Moreover, many studies show that high-credibility influencers who align with their followers’ social identities can significantly boost social satisfaction. Chen, Su, and Widjaja^73^ examined how social satisfaction enhances consumers’ willingness to buy through social interactions on Facebook. Brodie et al.^74^ found that social satisfaction affects brand loyalty and word-of-mouth by enhancing user engagement. Numerous studies have shown that social satisfaction between followers and influencers positively influences purchase intention. Shiau et al.^75^ applied social exchange theory to explain the parasocial relationship in terms of cost and reward, finding that maximizing social satisfaction can lead to higher purchase intention. Based on social comparison theory, H7 is proposed:

**H7:** Customer’s social satisfaction would positively affect their final purchase intention and mediates the relationship between influencer characteristics and purchase intention.

### Customer’s knowledge on the product

Campbell and Keller^76^ demonstrated that brand familiarity significantly moderates the effects of advertising repetition on consumer responses, suggesting that prior knowledge shapes information processing and attitude formation. In the context of social media marketing, Lim et al.^77^ found that consumers’ pre-existing brand attitudes influence their perceptions of social media influencers, highlighting the relevance of prior brand-related cognitions in shaping consumer responses to marketing messages. Furthermore, Chae et al.^78^ examined how brand familiarity affects consumer reactions to social media marketing for luxury brands, revealing that prior knowledge modulates the effectiveness of word-of-mouth campaigns. These findings suggest that consumers’ pre-existing knowledge may play a crucial role in how they interpret and respond to influencer marketing efforts. By incorporating consumers’ product knowledge as a moderating variable, this study aims to address this critical gap and provide a more nuanced understanding of the mechanisms underlying influencer marketing effectiveness. Based on attribution theory, H8 is proposed:

**H8:** Customer’s knowledge on the product will moderate the level of their final purchase intention.

**Fig. 2:**
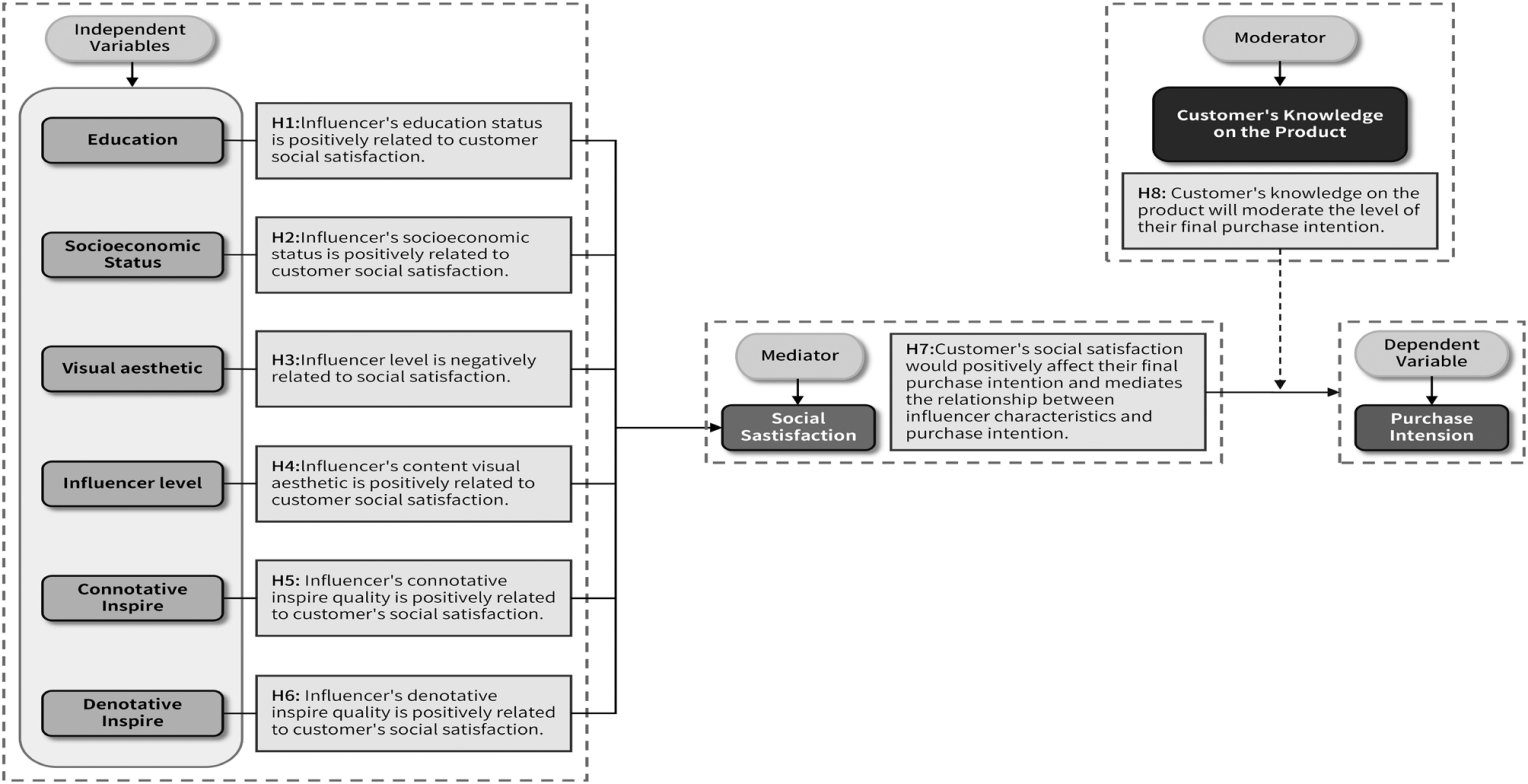
Conceptual model.

The conceptual model of this study is rooted in the theoretical framework of attribution theory. We posit that consumers’ perceptions of influencer characteristics trigger a series of attribution processes, which, in turn, affect their social satisfaction and purchase intentions. Specifically, an influencer’s educational background and socioeconomic status may be perceived as signals of ability and credibility, leading consumers to attribute the influencer’s success to internal factors. This attribution process is expected to positively impact social satisfaction. Meanwhile, visual aesthetics and content inspiration may be seen as manifestations of effort, prompting consumers to attribute positive experiences to the influencer’s professionalism, thereby enhancing social satisfaction. Based on the proposed hypotheses, a conceptual model is presented below in Figure 2.

## Methodology

**Table 1:** Operational Definitions of Constructs.

Construct	Definition	Measurement
Education (EDU)	The influencer’s educational level and its perceived impact on their expertise	7-point Likert scale assessing audience perceptions of the influencer’s educational background and its relevance to their content
Socioeconomic Status (SOC)	The perceived economic and social standing of the influencer	7-point Likert scale measuring audience perceptions of the influencer’s lifestyle, income level, and social influence
Visual Aesthetic (VIS)	The perceived attractiveness and quality of the visual elements in the influencer’s content	7-point Likert scale rating aspects such as color harmony, composition, and overall visual appeal
Influencer Level (LEV)	The perceived reach and impact of the influencer based on their follower count and engagement rates	Categorized into nano (1,000–10,000 followers), micro (10,000–100,000 followers), and macro ($>100,000$ followers) levels; perceptions measured on a 7-point Likert scale
Connotative Inspire (CON)	The emotional and value-based impact of the influencer’s content	7-point Likert scale assessing the audience’s emotional resonance and perceived alignment with the influencer’s implied values
Denotative Inspire (DEN)	The direct, literal impact of the influencer’s product descriptions and demonstrations	7-point Likert scale measuring the clarity, informativeness, and persuasiveness of the influencer’s explicit content
Social Satisfaction (SSF)	The emotional and psychological fulfillment derived from interactions with the influencer	7-point Likert scale measuring feelings of connection, belonging, and alignment with the influencer’s persona
Customers’ Knowledge (CKP)	The level of prior knowledge about the product being promoted	7-point Likert scale assessing self-reported familiarity with the product’s features, uses, and market positioning
Purchase Intention (PI)	The likelihood of the audience to purchase the promoted product	7-point Likert scale measuring the strength of intention to buy the product based on the influencer’s recommendation

### Instrument development

The instrument design used a questionnaire to achieve the goal of model validation. The respondents to the questionnaire are social media users who follow influencers. Reliability and validity are assessed using SPSS. Table 1 shows the operational definitions of each factor. It should be noted that the instrument design was reviewed by five experts from both academia and industry for content validity, and all items were validated according to the I-CVI score^79^. Nine factors that may affect customer purchase behaviour were identified from the previous literature review and research gaps. Each factor is measured with three questions.

### Data collection

In this study, 416 samples were collected, all from followers of entertainment-type influencers; those who did not meet this criterion were not permitted to complete the questionnaire. Fifty-three samples were excluded as invalid due to a short response time (less than 100 seconds) or lack of engagement. A total of 363 samples were validated for analysis.To ensure the adequacy of our sample size (N=363), we conducted an a priory power analysis using G*Power 3.1 software. Our actual sample size substantially exceeds the minimum threshold indicated by the power analysis, ensuring sufficient statistical power for the analysis.Most respondents were aged 26–30 years (27.3%) and had a bachelor’s degree. Females accounted for 52.1% and males for 47.9% of the sample. In terms of experience with influencers, 34.7% of respondents had 1–3 years of exposure, while 23.7% had one year or less of experience with influencer marketing. As required by the study design, 100% of respondents were followers of entertainment-type influencers.The focus on entertainment-type influencers was strategically chosen for several reasons. From a theoretical perspective, entertainment influencers provide an ideal context for studying attribution theory, as their content blends personal characteristics with commercial messages. In addition, the entertainment context allows for a clearer examination of both denotative and connotative inspiration effects. Visual aesthetics is a key variable in our model. It plays a particularly crucial role in entertainment content. From a market perspective, entertainment constitutes one of the largest and most commercially significant segments in influencer marketing.

This study was conducted in full compliance with relevant guidelines and regulations for human subject research. The research protocol and survey instruments were reviewed and approved by the Research Ethics Committee of Universiti Teknologi Malaysia (UTM). Prior to data collection, all participants were provided with a detailed informed consent statement outlining the study’s purpose, the voluntary nature of participation, and data protection measures. Written informed consent was obtained from all subjects and/or their legal guardians. Data collection was conducted through the online platform Questionnaire Star, with strict confidentiality measures implemented to protect participants’ privacy and personal information. All collected data were anonymized, securely stored in accordance with data protection protocols, and used exclusively for research purposes.

### Data analysis of measurement model

The measurement model demonstrates good convergent and discriminant validity. Common method bias was measured using Harman’s single-factor criterion^80^. The first component variance (unrotated) was 14.85% (<50%). Furthermore, the analysis revealed variance inflation factors (VIF) below 2, indicating that the data were free of multicollinearity and confirming the absence of common method bias^81^. The factor loadings exceeded 0.5, with average variance extracted (AVE) values ranging from 0.52 to 0.82 and composite reliability (CR) values from 0.74 to 0.91, indicating acceptable item-construct associations, sufficient variance capture, and strong internal consistency. The Fornell-Larcker criterion confirmed discriminant validity, with the square root of each construct’s AVE exceeding the correlations with other constructs. However, the Education and Socioeconomic variables, calculated under the same dimension, potentially overlapped. To address this, we combined these into a single construct, resulting in an eight-latent-variable model for subsequent analysis.

### Path analysis of structural model

#### The significance and relevance of the structural model relationships

Table 2 shows the path coefficient of each factors. Path coefficient analysis reveals varying impacts of influencer characteristics on Social Satisfaction (SSF) and Purchase Intention (PI). Socioeconomic status (SOC, $\beta = 0.166$, S.E. $= 0.075$), Visual Aesthetic (VIS, $\beta = 0.250$, S.E. $= 0.079$), and Denotative Inspire (DEN, $\beta = 0.247$, S.E. $= 0.095$) show significant positive relationships with SSF. Conversely, Influencer Level (LEV, $\beta = 0.059$, S.E. $= 0.065$, $p = 0.366$) and Connotative Inspire (CON, $\beta = -0.106$, S.E. $= 0.071$) demonstrate non-significant relationships with SSF. Notably, SSF strongly influences Purchase Intention (PI, $\beta = 0.344$, S.E. $= 0.098$).

The paths LEV to SSF and CON to SSF are rejected with t-values of 0.904 and −1.486 respectively (both $< 1.96$, $p> 0.05$). These results were obtained using MPLUS Version 8.0, which calculates two-tailed p-values by default.

These findings highlight the significant role of certain influencer characteristics, particularly socioeconomic status, visual aesthetics, and denotative inspiration, in shaping customer perceptions and purchase intentions. However, influencer level and connotative inspiration appear to have less impact. This analysis contributes to our understanding of key factors in influencer marketing effectiveness.

**Table 2:** Path statistics.

Path with SSF	Estimate	S.E.(Standard	Est./S.E.	Two-Tailed
	(Path coef.)	error)	($t\text {-value}$)	P Value
SOC	0.166	0.075	2.220	0.026
VIS	0.250	0.079	3.151	0.002
LEV	0.059	0.065	0.904	0.366
CON	−0.106	0.071	−1.486	0.137
DEN	0.247	0.095	2.613	0.009
**PI Path with SSF**	0.344	0.098	3.507	0.000

#### Mediation effect analysis

Table 3 presents the mediation analysis results. The ‘c (Total effect)’ column shows the overall impact of independent variables on dependent variables, such as the socioeconomic status factor’s total effect of 0.127 on social satisfaction and purchase intention. The ‘C($p\text {-value}$)’ column indicates the statistical significance of these relationships.

**Fig. 3:**
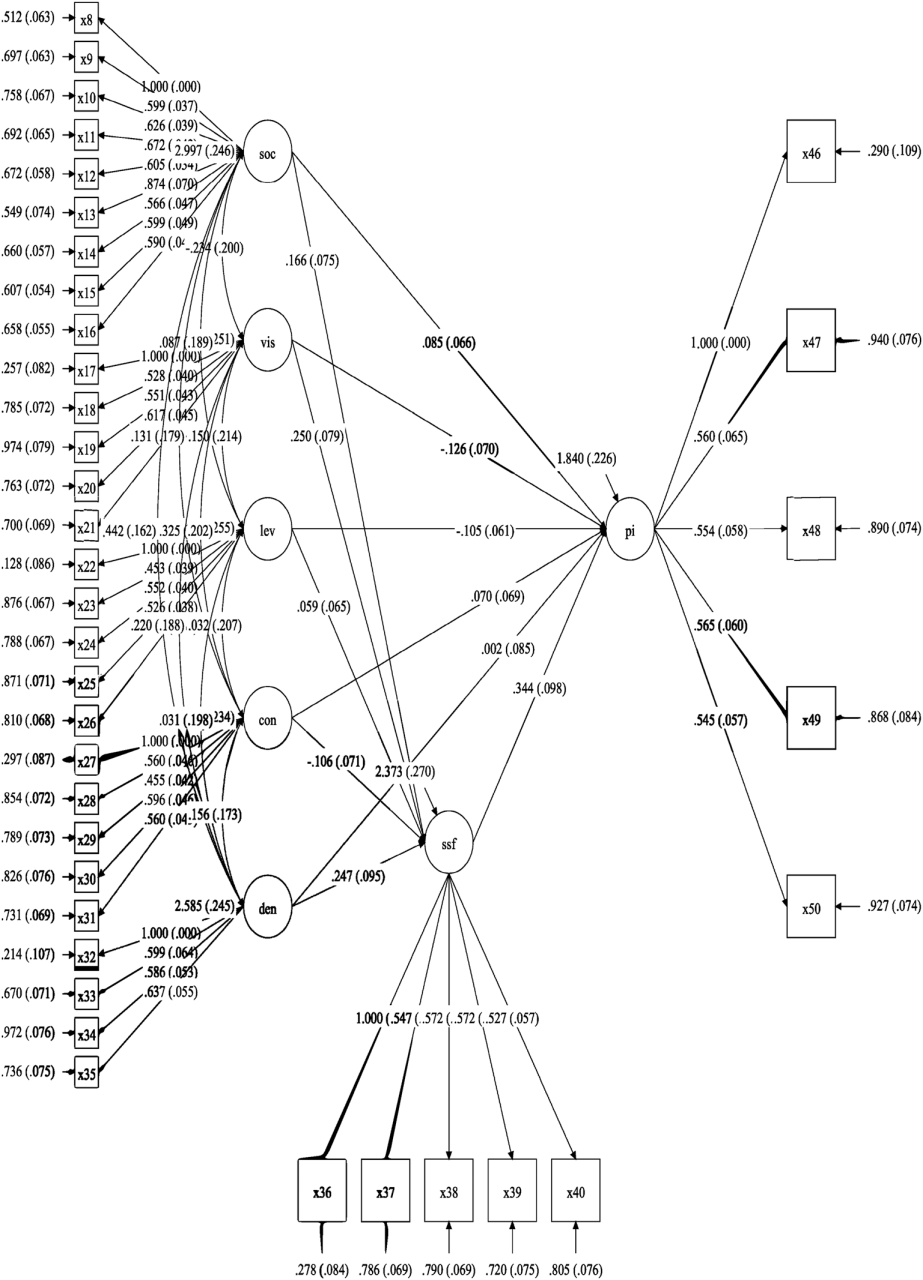
Mediator model.

Figure 3 illustrates the structural mediation equation model (SEM). It depicts how multiple observed variables (rectangular boxes, e.g., x1, x2,..., x6) indirectly influence other observed variables (e.g., SOC, LEV, CON), which in turn affect variables like PI. Arrows represent relationships between variables, with path coefficients and standard errors (in parentheses) displayed.

**Table 3:** Mediation statistics.

Mediation Path	c (Total effect	C(P-value)	a	a(p-value)	b	b(p-value)	a*b value)	a*b (P-value)	c’ (Direct effect	C’(P value)	Conclusion
Socioeconomic_status $=>$Social_satisfaction $=>$Purchase_intention	0.127	0.103	0.166	0.026	0.344	0.000***	0.057	0.055	0.085	0.198	Full mediation
Visual_aesthetic $=>$Social_satisfaction $=>$Purchase_intention	−0.051	0.544	0.250	0.002	0.344	0.000***	0.086	0.038	−0.126	0.072	Competitive mediation
Influencer_level $=>$Social_satisfaction $=>$Purchase_intention	−0.065	0.346	0.059	0.366	0.344	0.000***	0.020	0.419	−0.105	0.083	No significant mediation
Connotative_inspire $=>$Social_satisfaction $=>$Purchase_intention	0.050	0.506	−0.106	0.137	0.344	0.000***	−0.036	0.181	0.070	0.315	No significant mediation
Denotative_inspire $=>$Social_satisfaction $=>$Purchase_intention	0.066	0.448	0.247	0.009	0.344	0.000***	0.085	0.081	0.002	0.980	Partial mediation

The analysis reveals various mediation levels: full mediation, competitive mediation, no significant mediation, and partial mediation, based on the total effect, direct effect, and $p\text {-values}$. These findings enhance our understanding of the factors influencing social satisfaction and purchase intention in the context of influencer marketing.

### Moderation effect analysis

**Fig. 4:**
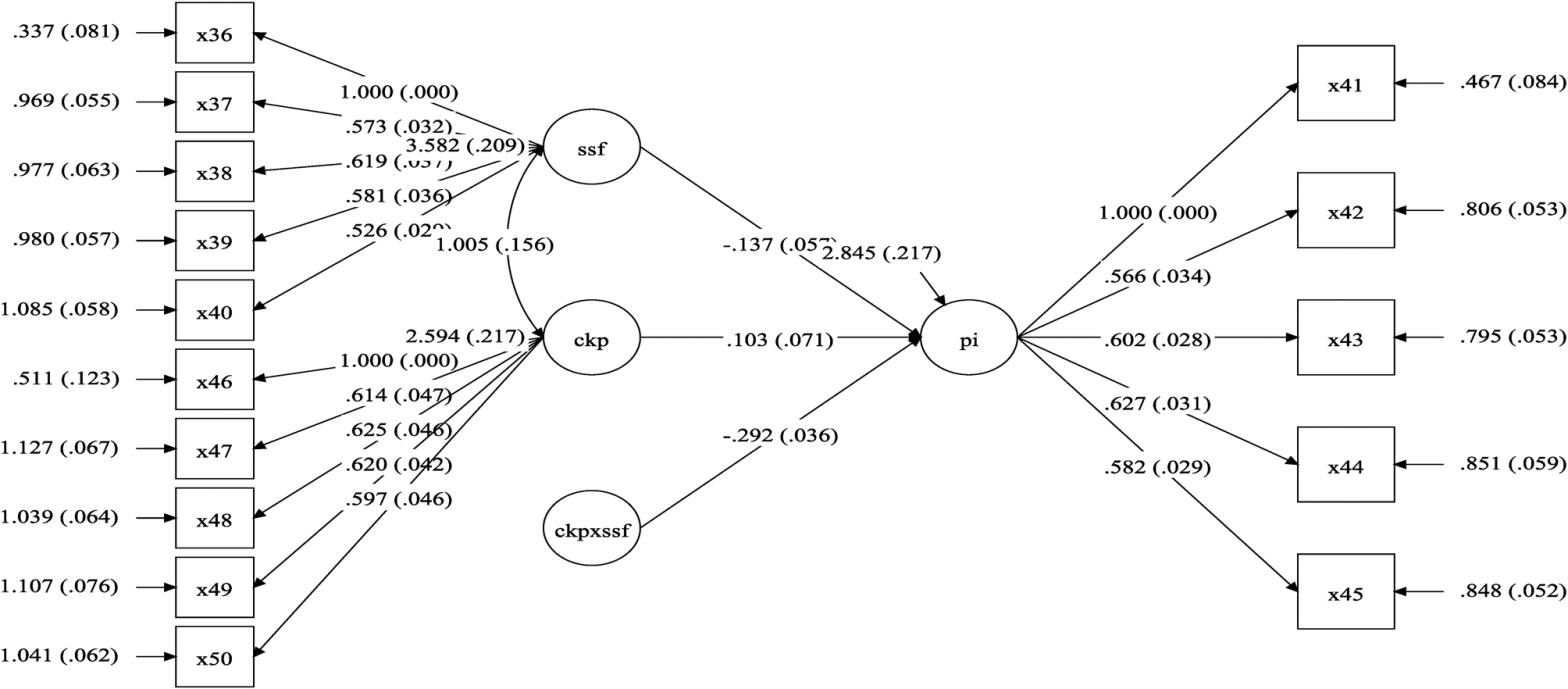
Moderator model.

Figure 4 shows the moderator model for H8; the parameters are the parameter estimator (outside parentheses)and standard error(inside parentheses). It also proved that the moderator effect is negative at −0.292. The interaction term (CPK$\times$SSF) showed in Table 4 presents a significant negative moderation effect (t = −8.062, p $< 0.001$).

**Table 4:** Moderate statistics.

PI on item	$t\text {-value}$	P value
CPK$\times$SSF	−8.062	0.000

### Hypothesis results

table 5 shows hypothesis testing, including the $t\text {-value}$ and $p\text {-value}$, and the number of bootstraps draws is 1000. The result shows that 4 hypotheses are supported out of 6 hypotheses, excluding the moderator and elimination of H1. The details are presented below, which are respectively the Socioeconomic status (H2), Visual aesthetics (H3), Influencer level (H4), Connotative inspire (H5), Denotative Inspire (H6), Social satisfaction (H7). It could be seen that H2, H3, H6, and H7 are supported, while H4 and H5 are rejected.

**Table 5:** Discussion of hypothesis.

Hypotheses	$t\text {-value}$	$p\text {-value}$	Result	Description
H2	2.22	0.026	Support	Given that Education and Socioeconomic status are combined in the PCA step, H1 & H2 are combined as H2.
				H2 stands for Influencer’s socioeconomic status is positively related to customer social satisfaction.
				H2 is supported as the $$t\text {-value}$$$$= 2.220>1.96$$ and $$p\text {-value}$$$$= 0.026 < 0.05$$
H3	3.151	0.002	Support	H3 stands for Influencer’s content visual aesthetics is positively related to customer social satisfaction.
				H3 is supported as the t- value $= 3.151>1.96$ and $p\text {-value} = 0.002 < 0.05$
H4	0.904	0.366	Reject	H4 stands for Influencer level and is negatively related to social satisfaction.
				H4 is rejected as the t- value $= 0.904< 1.96$ and $p\text {-value}= 0.366>0.05$
H5	−1.486	0.137	Reject	H5 stands for Influencer’s connotative inspire quality is positively related to customer’s social satisfaction.
				H5 is rejected as the t- value $= -1.486< 1.96$ and $p\text {-value}= 0.137>0.05$
H6	2.613	0.009	Support	H6 stands for Influencer’s denotative inspire quality is positively related to customer’s social satisfaction.
				H6 is supported as the t- value $= 2.613>1.96$ and $p\text {-value} = 0.009 < 0.05$
H7	3.507	0	Support	H7 stands for customer’s social satisfaction would positively affect their final purchase intention and mediates the relationship between influencer characteristics and purchase intention.
				H7 is supported as the t- value $= 3.507>1.96$ and $p\text {-value} = 0.000 < 0.05$
H8	−8.062	0	Support	H8 stands for customers’ knowledge on the product will moderate the level of their final purchase intention.
				The food group H8 is supported as $$t\text {-value}$$$$=| -8.062|> 1.96$$ and $$p\text {-value} = 0.000 < 0.05$$

### Model performance

As for the model performance, there are several statistics indicators for evaluation, such as Chi-Square, TLI, CFI and RMSEA. Table 6 shows each statistic indicator details below. Based on all key fit indices ($X^{2}$/df=1.127, TLI=0.906, CFI=0.914, RMSEA=0.019) meeting or significantly exceeding accepted standards, the model is shown to have excellent fit.

**Table 6:** Model performance.

Model fit indicator	Model performance	Acceptable values
$\chi ^{2}$	726.009	
$X^{2}$/df(Chi-squared ratio)	1.127	<3(Tomarken and Waller 2005)
TLI(NNFI;Tucker-Lewis index)	0.906	$>=$.90(Bentler and Bonett 1980)
CFI(Comparative Fit Index)	0.914	$>=$.90(Bentler 1990)
RMSEA(Root Mean Square Error of Approximation)	0.019	<.08(Hair Jr 2006)

## Discussion of hypothesis

### Education and socioeconomic status

**H1:Influencer’s education status is positively related to customer social satisfaction.**

**H2: Influencer’s socioeconomic status is positively related to customer social satisfaction.**

As illustrated in the formal section, this hypothesis is combined with socioeconomic status(H2), as the PCA results show they are in the same dimension. The combination of education and socioeconomic status into a single construct aligns with classical sociological theories^82^ that posit a strong correlation between educational attainment and socioeconomic position. Our findings support this relationship in the context of influencer marketing, suggesting that followers perceive influencers with higher education and socioeconomic status as more credible and satisfying.

For this hypothesis H2, the $t\text {-value}$ and $p\text {-value}$ results are supportive($t\text {-value} =2.220$, $p\text {-value} = 0.026$). Figure 3 shows that for Socioeconomic status variables, the estimator (path coefficient) is 0.166 towards social satisfaction, and the estimator towards Purchase intention is 0.085 ($p\text {-value} = 0.0$), indicating that socioeconomic status has a significant effect towards social satisfaction rather than the effect on purchase intention directly. This result extends the work of Black et al.^53^ and Oyibo, Adaji, and Vassileva^54^ by demonstrating that the positive effect of education on credibility applies not only to brand advertising but also to individual influencers. The significant positive relationship between this combined factor and social satisfaction suggests that followers may view highly educated, socioeconomically advantaged influencers as more knowledgeable and trustworthy sources of information.

However, this finding also raises questions about potential biases in influencer marketing. Future research should explore whether this preference for high-status influencers might reinforce existing social inequalities or limit the diversity of voices in social media marketing.

### Influencer level

**H3: Influencer level is negatively related to social satisfaction.**

For this hypothesis, the $t\text {-value}$ and $p\text {-value}$ results are rejective integrally ($t\text {-value} =0.904$, $p\text {-value} = 0.366$). The hypothesis proposed that influencer level will negatively affect social satisfaction. Contrary to our hypothesis, the results showed no significant relationship between influencer level and social satisfaction. This unexpected finding challenges the assumption that larger follower counts necessarily lead to greater influence or satisfaction.

This result may reflect a changing landscape in influencer marketing, where authenticity and engagement are becoming more valued than sheer reach. It aligns with recent studies suggesting that micro-influencers can be more effective in niche markets^60^. The lack of a significant relationship might indicate that followers prioritize content quality and relatability over an influencer’s popularity.

This finding has important implications for brands and marketers, suggesting that influencer selection should not be based solely on follower count. Future research could explore the potential non-linear relationships between influencer level and various outcome variables, as well as investigate the factors that make smaller influencers effective in certain contexts.

### Visual aesthetics

**H4:Influencer’s content visual aesthetic is positively related to customer social satisfaction.** For the H4, it is supported by the t- value =$3.151> 1.96$ and $p\text {-value} = 0.002 < 0.05$, indicating that the influencers’ content visual aesthetic is crucial for their followers’ social satisfaction. Influencers should form a typical style for their social media platforms to attract more followers.The strong positive relationship between visual aesthetics and social satisfaction underscores the importance of visual content in influencer marketing. This finding aligns with previous research in e-commerce contexts^63,64^ but extends it to the realm of social media influencers.

The significant impact of visual aesthetics on social satisfaction suggests that followers’ engagement goes beyond mere information processing; it involves an aesthetic experience that contributes to overall satisfaction. This finding has important implications for influencer marketing strategies, indicating that investments in high-quality visual content may yield substantial returns in terms of follower satisfaction and engagement. Future research could explore the specific elements of visual aesthetics (e.g., color schemes, composition, authenticity) that most strongly influence social satisfaction in different cultural contexts or across various social media platforms.

### Connotative inspire and denotative inspire

**H5: Influencer’s connotative inspire quality is positively related to customer’s social satisfaction. **

**H6: Influencer’s denotative inspire quality is positively related to customer’s social satisfaction. **

For H5, the $t\text {-value}$ and $p\text {-value}$ results are rejective($t\text {-value} =-1.486$, $p\text {-value} = 0.137$). For H6, the $t\text {-value}$ and $p\text {-value}$ results are rejective($t\text {-value}$ =2.613, $p\text {-value} = 0.009$). The contrasting results for connotative and denotative inspire qualities provide intriguing insights into the nature of influencer-follower relationships. The rejection of H5 and support for H6 suggest that followers value clear, factual information (denotative content) over emotional or symbolic messaging (connotative content) in influencer marketing.

This finding challenges some traditional marketing approaches that emphasize emotional appeals. In the context of influencer marketing, it appears that followers appreciate straightforward, informative content more than attempts at emotional manipulation. This aligns with recent trends towards transparency and authenticity in marketing^83^.

The preference for denotative content may reflect a more sophisticated, critical audience that is wary of overly emotional marketing tactics. It suggests that influencers and brands might benefit from focusing on providing clear, factual information about products or services rather than relying heavily on emotional appeals.

Future research could explore how this preference for denotative content varies across different product categories, cultural contexts, or demographic groups. It would also be valuable to investigate how a balance of denotative and connotative content might optimize influencer effectiveness.

### Social satisfaction with purchase intention

**H7:Customer’s social satisfaction would positively affect their final purchase intention and mediates the relationship between influencer characteristics and purchase intention.**

For this hypothesis, the $t\text {-value}$ and $p\text {-value}$ results are supportive integrally ($t\text {-value} =3.507$, $p\text {-value} = 0.000$). Figure 3 shows that the estimator (path coefficient) for the social satisfaction variables is 0.344 toward the purchase intention. The strong support for H7 underscores the critical role of social satisfaction in the influencer marketing process. This finding extends previous research on opinion leadership in social media^84,85^ by demonstrating that the satisfaction derived from social interactions with influencers can significantly impact purchase decisions.

The mediating role of social satisfaction suggests that the effect of influencer characteristics on purchase intention is not direct, but rather operates through the cultivation of a satisfying social experience. This highlights the importance of relationship-building in influencer marketing, moving beyond mere product promotion to creating engaging, satisfying social interactions.

This finding has important implications for influencer marketing strategies. It suggests that brands and influencers should focus not just on product-related content, but on creating overall satisfying experiences for followers. Future research could explore the specific components of social satisfaction in the influencer-follower relationship and how these can be optimized to enhance marketing effectiveness.

### Customer’s knowledge on the product

**H8: Customer’s knowledge on the product will moderate the level of their final purchase intention.**

For this hypothesis, the $t\text {-value}$ and $p\text {-value}$ results are supportive integrally($t\text {-value} =-8.062$, $p\text {-value} = 0.000$), which indicates that the moderator effect is verified. The support for H8 introduces an important nuance to our understanding of influencer marketing effectiveness. The moderating role of customer knowledge suggests that the impact of influencer marketing may vary significantly depending on the audience’s pre-existing familiarity with the product.

This finding aligns with previous research on the role of prior knowledge in information processing and decision-making^76,77^. It extends these insights to the specific context of influencer marketing, suggesting that the effectiveness of influencer strategies may depend on the target audience’s level of product knowledge.

The practical implications of this finding are significant. It suggests that influencer marketing strategies should be tailored based on the target audience’s level of product knowledge. For audiences with high product knowledge, influencers might focus on providing in-depth, technical information. For less knowledgeable audiences, a focus on basic product features and benefits might be more effective.This could be explained that knowledgeable consumers typically possess more sophisticated information processing capabilities and established evaluation criteria. Their extensive product knowledge may lead them to rely more on objective product attributes rather than influencer recommendations, resulting in more calculated purchase decisions. In this regards, brands targeting knowledgeable consumers should focus on providing detailed product information and technical specifications rather than relying solely on influencer endorsements.

Future research could explore how different types of influencer content (e.g., tutorials, reviews, lifestyle integration) interact with varying levels of customer knowledge to affect purchase intentions. Additionally, investigating how influencers can effectively cater to audiences with diverse levels of product knowledge within the same campaign could provide valuable insights for marketing practitioners.

In conclusion, these findings contribute to a more nuanced understanding of influencer marketing dynamics. They highlight the complex interplay between influencer characteristics, content attributes, audience factors, and marketing outcomes. Future research should continue to explore these relationships across different cultural contexts, product categories, and social media platforms to further refine our understanding of effective influencer marketing strategies.

## Conclusion

### Summary of the findings

Using structural equation modeling (SEM) analysis, this study uncovers the intricate relationships between influencer characteristics, content attributes, and consumer purchase intentions, with social satisfaction serving as a critical mediating factor. These findings contribute to the existing literature in several ways. First, they reveal how influencer characteristics (such as socioeconomic status) and content attributes (such as visual aesthetics) influence purchase intentions through social satisfaction, offering a new perspective on the psychological mechanisms of influencer marketing. Second, the non-significant relationships between influencer level, connotative inspiration, and social satisfaction warrant deeper examination. In the Chinese social media landscape, where content consumption patterns are rapidly evolving, the traditional emphasis on influencer status may be giving way to more nuanced evaluations of content quality and authenticity. The preference for denotative over connotative content might reflect Chinese consumers’ growing sophistication and desire for practical, straightforward information rather than emotional appeals. In terms of platform dynamics, the entertainment-focused nature of the studied influencers may affect how followers process and value different content types. The fast-paced, short-form content characteristic of Chinese social platforms might favor clear, direct communication over subtle, emotional messaging. These findings suggest that brands and marketers should focus on content quality and authenticity rather than solely pursuing high follower counts. Moreover, this study, conducted within the context of the Chinese market, provides valuable insights into how cultural factors shape influencer marketing dynamics. For example, the significant impact of socioeconomic status on social satisfaction may reflect the emphasis on social status within Chinese society. Overall, these findings offer important implications for both the theory and practice of influencer marketing. They emphasize the complex interactions between influencer characteristics, content quality, and consumer factors, providing a foundation for developing more effective influencer marketing strategies. Future research could further explore variations in these relationships across different product categories and platforms, and how these insights can be leveraged to optimize the return on investment in influencer marketing.

#### Limitations of the study

One limitation of this study pertains to the data collection measurement. The use of a 7-point Likert scale format introduces potential biases, particularly social desirability bias, where respondents might adjust their answers to appear more socially acceptable. This may affect the accuracy of responses regarding purchase intentions. Future research could consider incorporating qualitative methods or open-ended questions to capture a more comprehensive understanding of participants’ perspectives and allow for a richer exploration of their attitudes and perceptions. While this study focused exclusively on entertainment-type influencers, this choice was deliberate and aligned with our research objectives. The entertainment sector provides an ideal context for examining our theoretical framework. However, we acknowledge that this focus may limit the generalizability of our findings. Future research could examine whether the relationships identified in this study manifest differently across other influencer categories as well as respondents, the demographic homogeneity and single-category focus may limit the generalizability of the findings across different age groups and influencer types.

In terms of research design, the selection of latent variables posed a challenge in this study. All nine factors were treated as latent variables with five measurement items each, resulting in a computationally intensive process when using MPLUS. To avoid the computational issue, the moderator model training process is conducted separately from the mediator test. What is more, future research could form the moderator factor as the binary variable in order to improve computational efficiency.

#### Suggestions for future research

Combining the limitations discussed before, future research should focus on the mentioned gap in the literature by jumping from a general view and specific aspects of personal factors in terms of influencer characteristics and content attributions. To address this, a series of sub-dimensions of influencer factors was listed in the study. For example, the relevancy, tone and style, and voice. Specifically, examining how different appearances or vocal styles contribute to influencer content affinity and examining their potential impact on customers’ perceptions would provide a valuable research pathway. What is more, research could investigate how variables such as education and financial status contribute to a customers’ impression of an influencer. Additionally, examining the influence of region and job origin on the perception of influencer credibility could also be a research pathway.

Besides, future research can further explore the application of attribution theory in different types of influencer marketing(fitness, beauty and etc.). Specifically, researchers can investigate how different types of attributions affect consumers’ acceptance of influencer content and their willingness to engage. Additionally, longitudinal studies can examine how consumers’ attribution processes change over time, particularly in the context of long-term interactions with a specific influencer. Finally, cross-cultural studies can test the differences in attribution processes across different cultural contexts, providing guidance for global influencer marketing strategies.

Moreover, with the development of digital technology, Influencer Marketing, Artificial Intelligence (AI) and Metaverse, social trust and transparency and psychological and behavioural sciences will be the future direction of research, such as understanding the psychological and behavioural changes of the user in the virtual space, and applying them to the development of marketing strategies.

## Data Availability

The datasets generated and analyzed for this study are not publicly available due to privacy and ethical considerations but are available from the corresponding author upon reasonable request.

## References

[1] Gelper, S., van der Lans, R. & van Bruggen, G. Competition for attention in online social networks: Implications for seeding strategies. *Management Science* **67**, 1026–1047 (2021).

[2] Varsamis, E. Are social media influencers the next-generation brand ambassadors? forbes, june 13 (2018).

[3] Yadav, M. & Rahman, Z. Influencers on social media: As references for consumers. *Journal of Retailing and Consumer Services* **64**, 102786 (2022).

[4] Abidin, C. & Brown, M. L. Social media influencers. *ResearchGate* (2020).

[5] Yodel, G. What is influencer marketing? *Huffington Post* (2017).

[6] Kim, D. H., Seely, N. K. & Jung, J. H. Rise of social media influencers as a new marketing channel: Focusing on the roles of psychological well-being and perceived social responsibility among consumers. *International Journal of Environmental Research and Public Health* **19**, 1922 (2022). 35206553 10.3390/ijerph19042362 PMC8872418

[7] China Internet Network Information Center. *The 46th statistical report on internet development in china* (Statistical Report, 2020).

[8] Statista Research Department. Penetration rate of social media in china 2020-2023 (2023).

[9] Hu, L., Min, Q., Han, S. & Liu, Z. Understanding followers’ stickiness to digital influencers: The effect of psychological responses. *International Journal of Information Management* **54**, 102169 (2020).

[10] Lou, C. & Yuan, S. Influencer marketing: how message value and credibility affect consumer trust of branded content on social media. *Journal of Interactive Advertising* **19**, 58–73 (2019).

[11] De Veirman, M. & Hudders, L. Influencer marketing: brand control, commercial orientation and post credibility. *Journal of Marketing Management* **36**, 1805–1831 (2020).

[12] Kannan, P. & Li, H. A. Digital marketing: A framework, review and research agenda. *International Journal of Research in Marketing* **33**, 22–45 (2016).

[13] Voramontri, D. & Klieb, L. Impact of social media on consumer behaviour. *International Journal of Information and Decision Sciences* **11**, 209–233 (2019).

[14] Jin, S. V., Muqaddam, A. & Ryu, E. Instafamous and social media influencer marketing. *Marketing Intelligence & Planning* (2019).

[15] Martínez-López, F. J., Anaya-Sánchez, R., Fernández Giordano, M. & Lopez-Lopez, D. Behind influencer marketing: key marketing decisions and their effects on followers’ responses. *Journal of Marketing Management* **36**, 579–607 (2020).

[16] Zhu, Y. & Chen, H. Social media marketing content on short video platforms: A study on tiktok. *Journal of Advertising* **50**, 89–103 (2021).

[17] Whetten, D. A. What constitutes a theoretical contribution?. *Academy of Management Review* **14**, 490–495. 10.5465/amr.1989.4308371 (1989).

[18] De Jans, S., Cauberghe, V. & Hudders, L. How an advertising disclosure alerts young adolescents to sponsored vlogs: The moderating role of a peer-based advertising literacy intervention through an informational vlog. *Journal of Advertising* **47**, 309–325 (2018).

[19] Djafarova, E. & Trofimenko, O. ‘instafamous’-credibility and self-presentation of micro-celebrities on social media. *Information, communication & society* **22**, 1432–1446 (2019).

[20] Friestad, M. & Wright, P. The persuasion knowledge model: How people cope with persuasion attempts. *Journal of consumer research* **21**, 1–31 (1994).

[21] Hwang, Y. & Jeong, S.-H. “this is a sponsored blog post, but all opinions are my own’’: The effects of sponsorship disclosure on responses to sponsored blog posts. *Computers in Human Behavior* **62**, 528–535 (2016).

[22] Jhawar, A., Varshney, S. & Kumar, P. Effects of sponsorship disclosures on social media influencer-user psychological contract violation: moderated mediation effects through the expectancy violations lens. *Journal of Consumer Marketing ahead-of-print*10.1108/JCM-05-2023-5687 (2024).

[23] Festinger, L. A theory of social comparison processes. *Human relations* **7**, 117–140 (1954).

[24] Kelley, H. H. Attribution theory in social psychology. *Nebraska symposium on motivation* **15**, 192–238 (1967).

[25] Folkes, V. S. The availability heuristic and perceived risk. *Journal of Consumer research* **15**, 13–23 (1988).

[26] Kim, A., Balasubramanian, S. K. & Fiore, A. M. Attribution modeling in digital advertising: An empirical study of the impact of digital sales channels. *Journal of Marketing Analytics* **4**, 87–101 (2016).

[27] Jhawar, A. et al. Can virtual influencers induce destination visit intentions? a mediation analysis journey through the uncanny valley. *Journal of Travel Research*10.1177/00472875241291144 (2024).

[28] Kumar, V., Rajan, B., Venkatesan, R. & Lecinski, J. Artificial intelligence in marketing: Systematic review and future research direction. *Journal of Interactive Marketing* **49**, 23–43 (2020).

[29] Kapitan, S. & Silvera, D. H. From digital media influencers to celebrity endorsers: attributions drive endorser effectiveness. *Marketing Letters* **27**, 553–567 (2016).

[30] Kim, A., Balachander, S. & Kannan, K. Attribution modeling in digital advertising: An empirical study of the impact of digital sales channels. *Journal of Marketing Research* **54**, 536–553 (2017).

[31] Li, H. & Kannan, P. K. Attributing conversions in a multichannel online marketing environment: An empirical model and a field experiment. *Journal of Marketing Research* **51**, 40–56 (2014).

[32] Abhishek, V., Fader, P. & Hosanagar, K. Media exposure through the funnel: A model of multi-stage attribution. *Available at SSRN 2158421* (2012).

[33] Berman, R. Beyond the last touch: Attribution in online advertising. *Marketing Science* **37**, 771–792 (2018).

[34] Anderl, E., Becker, I., Von Wangenheim, F. & Schumann, J. H. Mapping the customer journey: Lessons learned from graph-based online attribution modeling. *International Journal of Research in Marketing* **33**, 457–474 (2016).

[35] Ghose, A. & Todri-Adamopoulos, V. Toward a digital attribution model: Measuring the impact of display advertising on online consumer behavior. *MIS Quarterly* **40**, 889–910 (2016).

[36] Boerman, S. C. The effects of the standardized instagram disclosure for micro-and meso-influencers. *Computers in Human Behavior* **103**, 199–207 (2020).

[37] Casaló, L. V. & Escario, J.-J. Heterogeneity in the association between environmental attitudes and pro-environmental behavior: A multilevel regression approach. *Journal of Cleaner Production* **175**, 155–163 (2018).

[38] Torres, P., Augusto, M. & Matos, M. Antecedents and outcomes of digital influencer endorsement: An exploratory study. *Psychology & Marketing* **36**, 1267–1276 (2019).

[39] Chung-Wha Chloe, K. & Cuevas, L. M. *& Lim, H* (An exploratory study, 2019).

[40] Ki, C.-W.C., Cuevas, L. M., Chong, S. M. & Lim, H. Influencer marketing: Social media influencers as human brands attaching to followers and yielding positive marketing results by fulfilling needs. *Journal of Retailing and Consumer Services* **55**, 102133 (2020).

[41] Brown, T. A. & Moore, M. T. Confirmatory factor analysis. *Handbook of structural equation modeling* **361**, 379 (2012).

[42] Goodman, J. K., Cryder, C. E. & Cheema, A. Data collection in a flat world: The strengths and weaknesses of mechanical turk samples. *Journal of Behavioral Decision Making* **26**, 213–224 (2013).

[43] Mason, W. & Suri, S. Conducting behavioral research on amazon’s mechanical turk. *Behavior research methods* **44**, 1–23 (2012). 21717266 10.3758/s13428-011-0124-6

[44] Jang, W., Kim, J., Kim, S. & Chun, J. W. The role of engagement in travel influencer marketing: The perspectives of dual process theory and the source credibility model. *Current Issues in Tourism* **24**, 2416–2420 (2021).

[45] Sokolova, K. & Kefi, H. Instagram and youtube bloggers promote it, why should i buy? how credibility and parasocial interaction influence purchase intentions. *Journal of Retailing and Consumer Services* **53**, 101742 (2020).

[46] Kozinets, R. V., De Valck, K., Wojnicki, A. C. & Wilner, S. J. Networked narratives: Understanding word-of-mouth marketing in online communities. *Journal of marketing* **74**, 71–89 (2010).

[47] De Veirman, M., Cauberghe, V. & Hudders, L. Influencer marketing: The impact of disclosing sponsorship compensation justification on sponsored content effectiveness. *Journal of Marketing Communications* **23**, 109–126 (2017).

[48] Khamis, S., Ang, L. & Welling, R. Self-branding’,micro-celebrity’and the rise of social media influencers. *Celebrity studies* **8**, 191–208 (2017).

[49] Xiao, M., Wang, R. & Chan-Olmsted, S. Factors affecting youtube influencer marketing credibility: a heuristic-systematic model. *Journal of media business studies* **15**, 188–213 (2018).

[50] Kelman, H. C. Compliance, identification, and internalization three processes of attitude change. *Journal of conflict resolution* **2**, 51–60 (1958).

[51] Kim, A. J. & Ko, E. Do social media marketing activities enhance customer equity? an empirical study of luxury fashion brand. *Journal of Business Research* **65**, 1480–1486. 10.1016/j.jbusres.2011.10.014 (2012).

[52] Alba, J. W. & Hutchinson, J. W. Dimensions of consumer expertise. *Journal of Consumer Research* **13**, 411–454. 10.1086/209080 (1987).

[53] Black, A., Fernandez, M. A., Desroches, S. & Raine, K. D. Education matters: Certified health professionals have higher credibility than non health professionals on instagram. *Alberta Academic Review* **2**, 11–12 (2019).

[54] Oyibo, K., Adaji, I. & Vassileva, J. Mobile web design: the effect of education on the influence of classical and expressive aesthetics on perceived credibility (2019).

[55] Khan, A., Rizvi, S. A. R., Ali, M. & Haroon, O. A survey of islamic finance research-influences and influencers. *Pacific-Basin Finance Journal* **69**, 101437 (2021).

[56] Hati, S. R. H. & Idris, A. Antecedents of customers’ intention to support islamic social enterprises in indonesia: The role of socioeconomic status, religiosity, and organisational credibility. *Asia Pacific Journal of Marketing and Logistics* (2014).

[57] Shin, E. & Lee, J. E. What makes consumers purchase apparel products through social shopping services that social media fashion influencers have worn?. *Journal of Business Research* **132**, 416–428 (2021).

[58] Lee, J. E. & Watkins, B. Youtube vloggers’ influence on consumer luxury brand perceptions and intentions. *Journal of Business Research* **69**, 5753–5760 (2016).

[59] Ali, N., Tretiakov, A. & Whiddett, D. A content validity study for a knowledge management systems success model in healthcare. *JITTA: Journal of Information Technology Theory and Application* **15**, 21 (2014).

[60] Kay, S., Mulcahy, R. & Parkinson, J. When less is more: the impact of macro and micro social media influencers’ disclosure. *Journal of Marketing Management* **36**, 248–278 (2020).

[61] Jin, S. V., Ryu, E. & Muqaddam, A. I trust what she’s# endorsing on instagram: moderating effects of parasocial interaction and social presence in fashion influencer marketing. *Journal of Fashion Marketing and Management: An International Journal* (2021).

[62] De Jans, S., Van de Sompel, D., De Veirman, M. & Hudders, L. # sponsored! how the recognition of sponsoring on instagram posts affects adolescents’ brand evaluations through source evaluations. *Computers in Human Behavior* **109**, 106342 (2020).

[63] Harris, L. C. & Goode, M. M. Online servicescapes, trust, and purchase intentions. *Journal of Services Marketing* (2010).

[64] Lorenzo-Romero, C., Constantinides, E. & Alarcón-del Amo, M.-d.-C. Web aesthetics effects on user decisions: impact of exposure length on website quality perceptions and buying intentions. *Journal of internet commerce* **12**, 76–105 (2013).

[65] Zhang, J., Wei, W. & Wang, C. Effects of psychological interventions for patients with systemic lupus erythematosus: a systematic review and meta-analysis. *Lupus* **21**, 1077–1087 (2012). 22570339 10.1177/0961203312447667

[66] Bharti, M., Suneja, V. & Chauhan, A. K. The role of socio-psychological and personality antecedents in luxury consumption: a meta-analytic review. *International Marketing Review* (2021).

[67] Braatz, L. *# Influencer marketing on instagram: consumer responses towards promotional posts: the effects of message sidedness*. Master’s thesis, University of Twente (2017).

[68] Džanić, M. The semiotics of contemporary advertising messages: Decoding visuals. *Jezikoslovlje* **14**, 475–485 (2013).

[69] Puntoni, S., Schroeder, J. E. & Ritson, M. Meaning matters. *Journal of Advertising* **39**, 51–64 (2010).

[70] Kleine, R. E. III. & Kernan, J. B. Contextual influences on the meanings ascribed to ordinary consumption objects. *Journal of Consumer Research* **18**, 311–324 (1991).

[71] Mick, D. G. & Politi, L. G. Consumers’ interpretations of advertising imagery: A visit to the hell of connotation. *ACR Special Volumes* (1989).

[72] Lin, K.-Y. & Lu, H.-P. Why people use social networking sites: An empirical study integrating network externalities and motivation theory. *Computers in human behavior* **27**, 1152–1161 (2011).

[73] Chen, J. V., Su, B.-C. & Widjaja, A. E. Facebook c2c social commerce: A study of online impulse buying. *Decision Support Systems* **83**, 57–69 (2016).

[74] Brodie, R. J., Hollebeek, L. D., Jurić, B. & Ilić, A. Customer engagement: Conceptual domain, fundamental propositions, and implications for research. *Journal of service research* **14**, 252–271 (2011).

[75] Shiau, W.-L. & Luo, M. M. Factors affecting online group buying intention and satisfaction: A social exchange theory perspective. *Computers in Human Behavior* **28**, 2431–2444 (2012).

[76] Campbell, M. C. & Keller, K. L. Brand familiarity and advertising repetition effects. *Journal of consumer research* **30**, 292–304 (2003).

[77] Lim, X. J., Radzol, A. R. M., Cheah, J.-H. & Wong, M. W. The impact of social media influencers on purchase intention and the mediation effect of customer attitude. *Asian Journal of Business Research* **7**, 19–36 (2017).

[78] Chae, I., Stephen, A. T., Bart, Y. & Yao, D. Spillover effects in seeded word-of-mouth marketing campaigns. *Marketing Science* **36**, 89–104 (2017).

[79] Grant, J. S. & Davis, L. L. Selection and use of content experts for instrument development. *Research in nursing & health* **20**, 269–274 (1997). 9179180 10.1002/(sici)1098-240x(199706)20:3<269::aid-nur9>3.0.co;2-g

[80] Hair Jr, J. F., Black, W. C., Babin, B. J. & Anderson, R. E. *Multivariate Data Analysis* (Pearson Education Limited, 2014).

[81] Sinan, A. & Alkan, B. B. A useful approach to identify the multicollinearity in the presence of outliers. *Journal of Applied Statistics* **42**, 986–993. 10.1080/02664763.2014.993369 (2015).

[82] Ritzer, G. *Working: Conflict and change* (Transaction Publishers, 1975).

[83] Audrezet, A., de Kerviler, G. & Moulard, J. G. Authenticity under threat: When social media influencers need to go beyond self-presentation. *Journal of business research* **117**, 557–569 (2020).

[84] Cho, J. H., Lee, H. B. & Yoon, Y. Effects of local product perception and consumer ethnocentrism on consumer attitudes toward global products. *Asia Pacific Journal of Marketing and Logistics* **24**, 491–506 (2012).

[85] Park, Y.-G. & Kaye, B. The influence of product category and retail format on online store selection: A study of college students. *Journal of Electronic Commerce Research* **18**, 220–230 (2017).

